# Dual T-cell depletion with individually tailored anti-thymocyte globulin and attenuated dose of post-transplant cyclophosphamide in haploidentical peripheral stem cell transplantation

**DOI:** 10.1038/s41598-024-64361-5

**Published:** 2024-06-16

**Authors:** Dong Hyun Kim, Dong-Yeop Shin, Youngil Koh, Inho Kim, Sung-Soo Yoon, Ja Min Byun, Junshik Hong

**Affiliations:** 1 Department of Internal Medicine, Seoul National University Hospital, 101, Daehak-Ro, Jongro-Gu, Seoul, 03080 Republic of Korea; 2 Seoul National University Hospital, Biomedical Research Institute, Seoul, Korea; 3 Cancer Research Institute, Seoul National University College of Medicine, Seoul, Korea; 4 Cancer for Medical Innovation, Seoul National University Hospital, Seoul, Korea

**Keywords:** Haploidentical hematopoietic stem cell transplantation, Anti-thymocyte globulin, Post-transplant cyclophosphamide, Graft-versus-host disease, Bone marrow transplantation, Leukaemia

## Abstract

This study aimed to assess the efficacy of dual T-cell suppression using individually tailored doses of antithymocyte globulin (ATG) and attenuated dose of post-transplant cyclophosphamide (PTCy) in haploidentical hematopoietic stem cell transplantation (haplo-HSCT). We conducted a retrospective analysis of 78 adults with acute leukemia or myelodysplastic syndrome who underwent haplo-HSCT using intravenous busulfan and fludarabine conditioning. Thirty-two patients received attenuated ATG/PTCy, while 46 patients received ATG (7.5 mg/kg) as GVHD prophylaxis. The 100-day cumulative incidence of grade III-IV (9.7% vs. 32.4%, *P* = 0.018) acute GVHD, as well as 2-year moderate-severe chronic GVHD (13.9% vs. 43.9%, *P* = 0.018) in the ATG/PTCy group were significantly lower than those in the ATG group. The 2-year overall survival was comparable between the two groups. However, 2-year GVHD-free, relapse-free survival in the ATG/PTCy group was significantly higher compared to that in the ATG group (38.9% vs. 21.7%, *P* = 0.021). Moreover, during post-engraftment period, the ATG/PTCy group exhibited lower incidences of life-threatening bacterial (12.5% vs. 37%, *P* = 0.033) and viral infection (0% vs. 17.4%, *P* = 0.035) than the ATG group. In conclusion, the combination of individually tailored ATG and low-dose PTCy appears to be a promising strategy in haplo-HSCT.

## Introduction

Allogeneic hematopoietic stem cell transplantation provides a chance for cure in patients with hematologic malignancies, and familial haplo-identical hematopoietic stem cell transplantation (haplo-HSCT) is increasingly being performed in light of the donor’s immediate availability^1^. However, due to HLA mismatches^2,3^, graft-versus-host disease (GVHD) remains a significant challenge, and there is no consensus on the optimal GVHD prophylaxis regimen for haplo-HSCT.

Antithymocyte globulin (ATG) has been widely used in haplo-HSCT for GVHD prevention, offering advantages such as reduced rates of graft rejection and relapse^4–7^. However, the acute GVHD (aGVHD) rate did not drop below 40%, and the benefits are compromised by the high risk of infections, especially viral infections including cytomegalovirus (CMV) reactivation, due to delayed immune reconstitution^5,8–11^. On the other hand, Luznik et al. reported that high-dose post-transplant cyclophosphamide (PTCy) effectively reduces GVHD rates in haplo-HSCT^12^, with subsequent reports confirming its efficacy in allogeneic HSCT with mismatched donor^13,14^. However, challenges arise with high-dose PTCy, including excessive cytotoxicity leading to a risk of acute cardiotoxicity and potential attenuation of the graft-versus-leukemia effect^15,16^. Therefore, attempts have been made to address these issues by exploring the efficacy of low-dose PTCy^17^.

Considering the demand for optimal GVHD prophylaxis in haplo-HSCT, efforts have been made to combine low-dose PTCy and ATG to mitigate their respective disadvantages. This study aims to report on the efficacy of dual T-cell suppression using individually tailored ATG and attenuated doses of PTCy, comparing it to ATG alone as GVHD prophylaxis in haplo-HSCT for patients with acute leukemia and myelodysplastic syndrome (MDS).

## Patients and methods

### Participants and study design

This is a retrospective study of patients with acute leukemia and MDS who were over 18 years old and underwent haplo-HSCT following identical busulfan and fludarabine (BuFlu) conditioning between June 2017 and 2023 at Seoul National University Hospital, Seoul, South Korea. All patients received either ATG (ATG group) or a combination of ATG and PTCy (ATG/PTCy group) as T-cell depletion for GVHD prophylaxis. In the consideration of the choice between ATG and ATG/PTCy, some of the staff physicians implemented ATG/PTCy for research purposes, while the remainder adhered to the conventional institutional practice of applying ATG. Haploidentical donors were considered in cases where a matched donor was unavailable or when HSCT was urgently needed. In all patients, peripheral blood stem cells (PBSCs) were used as a graft source.

### Transplantation procedures

For the ATG group, busulfan 3.2 mg/kg and fludarabine 40 mg/m^2^ on days -6 through -3 were used as myeloablative conditioning (MAC), and busulfan 3.2 mg/kg on days -7 and -6 with fludarabine 30 mg/m^2^ on days -7 through -2 were used as reduced-intensity conditioning (RIC). ATG (rabbit, thymoglobulin; Sanofi-Aventis, Korea) 2.5 mg/kg on days -3 through -1, for a total of 7.5 mg/kg was administrated as GVHD prophylaxis. For the ATG/PTCy group, the MAC regimen was the same as in ATG group, while for the RIC, busulfan 3.2 mg/kg on days -6 and -5 with fludarabine 40 mg/m^2^ on days -6 through -3 were employed. ATG was given from days -3 through -1 and was adjusted per absolute lymphocyte count (ALC) on days -3 based on our previous study^18^: for ALC > 1,000/μl patients, total of 4.0 mg/kg was used; for ALC 500–1,000/μl, total of 3.5 mg/kg; and for ALC < 500/μl, total of 3.0 mg/kg was used. PTCy was administered at doses of 50 mg/kg on day 3 and 30 mg/kg on day 4 (Supplementary Fig. 1). In addition, calcineurin inhibitors were used to prevent GVHD. The choice of calcineurin inhibitors, either cyclosporine or tacrolimus, was left to the attending physician’s preference. Methotrexate (15 mg/m^2^ intravenous push on day 1 and 10 mg/m^2^ on day 3 and day 6) was considered in ATG group, and mycophenolate mofetil (15 mg/kg three times a day from days 5 through 30) was administered in ATG/PTCy group.

All patients received uniform prophylactic regimens; ciprofloxacin for gut decontamination, trimethoprim/sulfamethoxazole for Pneumocystis jirovecii, micafungin for fungus, and acyclovir for herpes simplex virus. The serum CMV polymerase chain reaction test was conducted twice weekly, and preemptive ganciclovir treatment was initiated in cases of CMV reactivation.

### Definitions and outcomes

The diagnosis of acute leukemia and MDS was made according to the revised 2016 WHO classification. The modified European Group for Blood and Marrow Transplantation (mEBMT) risk score was used to assess risks of HSCT^19^. The 2016 MAGIC criteria were used to grade aGVHD^20^. Chronic GVHD (cGVHD) was classified as mild, moderate, or severe according to the 2014 National Institutes of Health consensus criteria^21^. Neutrophil engraftment was defined as absolute neutrophil count (ANC) > 500/μl for 3 consecutive measurements. Platelet engraftment was defined as 3 consecutive measurements of > 20,000/μl without transfusion. Non-relapse mortality (NRM) was defined as death without progression of underlying disease. Relapse-free survival (RFS) was defined as the time from stem cell infusion to relapse or death from any cause. Overall survival (OS) was defined as the time from stem cell infusion to death of any cause. GVHD-free, relapse-free survival (GRFS) was defined according to Ruggeri et al. (ie, being alive with no grade 3–4 aGVHD, severe cGVHD, or relapse at any time point after HSCT). The cause of death was evaluated as primary disease, graft failure, GVHD, infection, and other based on a previous report^22^. Any bacterial, viral, and fungal Infection data were collected retrospectively until the patient’s death or last follow-up, using standardized definitions of severe infections after HSCT^23,24^. Infections requiring intensive care or vasopressor support were considered life-threatening infections.

### Statistical analysis

Differences between groups were assessed using a Student’s t-test or Wilcoxon rank-sum test for continuous variables, and chi-square test for categorical variables, as indicated. Median follow-up duration was calculated by the reverse Kaplan–Meier method. RFS, OS and GRFS were estimated by the Kaplan–Meier method and log-rank test was used for univariate comparisons of survival. Cumulative incidence curves were used in competing risk setting to calculate the probability of GVHD, relapse incidence and NRM. Univariate and multivariate Cox proportional hazards regression models were used to analyze GVHD and survival outcomes with type of GVHD prophylaxis, age, diagnosis, mEBMT score, conditioning intensity, amount of infused CD34 + cell, and HSCT timing. Multivariate analysis was performed using stepwise backward selection, and adjusted hazard ratios (HRs) with 95% confidence intervals (CIs) were calculated. All tests were 2-sided, and *P* values of < 0.05 were considered statistically significant. For all parts of statistical analyses, the statistical software ‘R’ version 4.3.1 (www.r-project.org) were used.

### Ethics declarations

This study was conducted according to Declaration of Helsinki and was approved by the Institutional Review Board of Seoul National University Hospital (IRB no. H-2207–083-1339). The requirement for informed consent was waived by the Institutional Review Board of Seoul National University Hospital due to the retrospective nature of the study.

## Results

### Patient and disease characteristics

A total of 78 patients were included in this study; 32 belonged to the ATG/PTCy group, while 46 were in the ATG group. Table 1 presents the baseline characteristics of the two groups. There were no differences in baseline characteristics, including disease status and infused CD34 cell dose, between the two groups.

**Table 1:** Baseline characteristics.

	All patients N = 78	ATG/PTCy N = 32	ATG N = 46	*P*-value
Age, year, median (range)	55 (21–69)	56 (23–68)	55 (21–69)	0.972
Age > 60 years, n (%)	25 (32.1)	11 (34.4)	14 (30.4)	0.904
Sex, n (%)				0.616
Male	40 (51.3)	18 (56.2)	22 (47.8)	
Female	38 (48.7)	14 (43.8)	24 (52.2)	
Diagnosis, n (%)				0.835
Myelodysplastic syndrome	23 (29.5)	9 (28.1)	14 (30.4)	
Acute myeloid leukemia	37 (47.4)	15 (46.9)	22 (47.8)	
Acute lymphoblastic leukemia	15 (19.2)	6 (18.8)	9 (19.6)	
Mixed-phenotype acute leukemia	3 (3.8)	2 (6.2)	1 (2.2)	
Disease status before HSCT, n (%)				0.480
CR1	28 (35.9)	14 (43.8)	14 (30.4)	
≥ CR2	20 (25.6)	14 (43.8)	13 (28.3)	
Not in remission	30 (38.5)	11 (34.4)	19 (41.3)	
Donor relationship, n (%)				0.533
Parent	7 (9.0)	3 (9.4)	4 (8.7)	
Child	46 (59.0)	21 (65.6)	25 (54.3)	
Sibling	25 (32.0)	8 (25.0)	17 (37.0)	
Number of HLA mismatched, n (%)				0.359
2	4 (5.1)	1 (3.1)	3 (6.5)	
3	20 (25.6)	6 (18.8)	14 (30.4)	
4	54 (69.2)	25 (78.1)	29 (63.0)	
DSA positive, n (%)	4 (5.1)	1 (3.1)	3 (6.5)	0.833
ABO matching, n (%)				1.000
Matched	30 (38.5)	12 (37.5)	18 (39.1)	
Mismatched	48 (61.5)	20 (62.5)	28 (60.9)	
CMV serostatus, n (%)				0.333
D-/R-	1 (1.3)	1 (3.1)	0 (0)	
D-/R +	8 (10.3)	5 (15.6)	3 (6.5)	
D + /R-	2 (2.6)	1 (3.1)	1 (2.2)	
D + /R +	67 (85.9)	25 (78.1)	42 (91.3)	
Modified EBMT score, n (%)				0.191
1–3	31 (39.7)	16 (50)	15 (32.6)	
4–6	47 (60.3)	16 (50)	31 (67.4)	
Conditioning intensity, MAC, n (%)	14 (17.9)	9 (28.1)	5 (10.9)	0.098
CD34 + cell, × 10^6^/kg, median (range)	5.06 (2.29–9.08)	5.14 (3.07–7)	5 (2.29–9.08)	0.348
Calcineurin inhibitor, n (%)				0.171
Cyclosporine	63 (80.8)	23 (71.9)	40 (87.0)	
Tacrolimus	15 (19.2)	9 (28.1)	6 (13.0)	
HSCT timing, n (%)				0.001
June 2017–June 2020	35 (44.9)	22 (68.8)	13 (28.3)	
July2020–June2023	43 (55.1)	10 (31.2)	33 (71.7)	

*ATG* antithymocyte globulin, *PTCy* post-transplant cyclophosphamide, *HSCT* hematopoietic stem cell transplantation, *CR* complete remission, *HLA* human leukocyte antigen, *DSA* donor-specific antibody, *CMV* cytomegalovirus, EBMT, European Society for Blood and Marrow Transplantation, *MAC* myeloablative conditioning.

### Transplantation outcomes

Neutrophil engraftment (93.8% vs. 95.7%) and platelet engraftment (90.6% vs. 91.3%) did not differ between the ATG/PTCy and ATG groups (Table 2). Regarding immune reconstitution, the ATG/PTCy group exhibited a trend toward faster recovery: time to neutrophil engraftment (median, 16 vs. 18 days, *P* = 0.002) and time to platelet engraftment (median, 15 vs. 18 days, *P* = 0.25). The incidence of both CMV antigenemia and disease was lower in the ATG/PTCy group, but the difference did not show significance (53.1% vs. 67.4%; *P* = 0.3, and 3.1% vs 19.6%; *P* = 0.073, respectively). Only two patients in the ATG group were found to have post-transplant lymphoproliferative disease as evidenced by biopsy.

**Table 2:** Post-transplantation outcomes.

	ATG/PTCy N = 32	ATG N = 46	*P*-value
Neutrophil engraftment, n (%)	30 (93.8)	44 (95.7)	1.000
Time to engraftment, day, median (range)	16 (13–30)	18 (12–46)	0.002
Platelet engraftment, n (%)	29 (90.6)	42 (91.3)	1.000
Time to engraftment, day, median (range)	15 (10–114)	18 (11–77)	0.250
CMV antigenemia, n (%)	17 (53.1)	31 (67.4)	0.300
Time to CMV antigenemia, day, median (range)	41 (15–864)	42 (3–516)	1.000
CMV disease, n (%)	1 (3.1)	9 (19.6)	0.073
Time to CMV disease, day, median (range)	112	56 (21–378)	
Cumulative incidence of outcome, % (95% CI)			
Acute GVHD at day 100			
Overall acute GVHD	38.6 (18.7–53.7)	54.0 (36.5–66.7)	0.075
Grade II-IV acute GVHD	22.1 (6.2–35.4)	42.9 (26.1–55.9)	0.046
Grade III-IV acute GVHD	9.7 (0.0–19.6)	32.4 (16.8–45.1)	0.018
Chronic GVHD at 2 years			
Overall chronic GVHD	46.3 (18.3–64.7)	57.6 (33.1–73.1)	0.330
Moderate-severe chronic GVHD	13.9 (0.0–27.6)	43.9 (21.4–60.0)	0.018
Severe chronic GVHD	6.3 (0.0–17.4)	28.4 (7.5–44.6)	0.072
Relapse at 2 years	46.4 (21.4–63.4)	54.2 (35.5–67.4)	0.300
NRM at 2 years	21.9 (2.3–37.6)	29.0 (10.8–43.4)	0.490
Other complications, n (%)			
Hemorrhagic cystitis	1 (3.1)	4 (8.7)	0.604
Post-transplantation lymphoproliferative disorder	0 (0)	2 (4.3)	0.641
Veno-occlusive disease	2 (6.2)	3 (6.5)	1.000
Death, n (%)	15 (46.9)	32 (69.9)	0.075
Cause of death, n (%)			0.508
Primary disease	6 (40)	17 (53.1)	
Graft failure	3 (20.0)	4 (12.5)	
GVHD-related	4 (26.7)	7 (21.9)	
Infection	1 (6.7)	4 (12.5)	
Others	1 (6.7)	0 (0)	

*ATG* antithymocyte globulin, *PTCy* post-transplant cyclophosphamide, *CMV* cytomegalovirus, *GVHD* graft-versus-host disease, *CI* cumulative incidence, *NRM* non-relapse mortality.

Up to December 2023, all of the patients were followed for a median of 38 months (95% CI, 29.4–44); the median follow-up time for ATG/PTCy and ATG groups were 29.4 and 43.3 months, respectively. During follow-up, 37 patients (47.4%) relapsed and 47 (60.3%) died. There was no difference between the groups in terms of causes of death.

### Graft-versus-host disease

**Figure 1:**
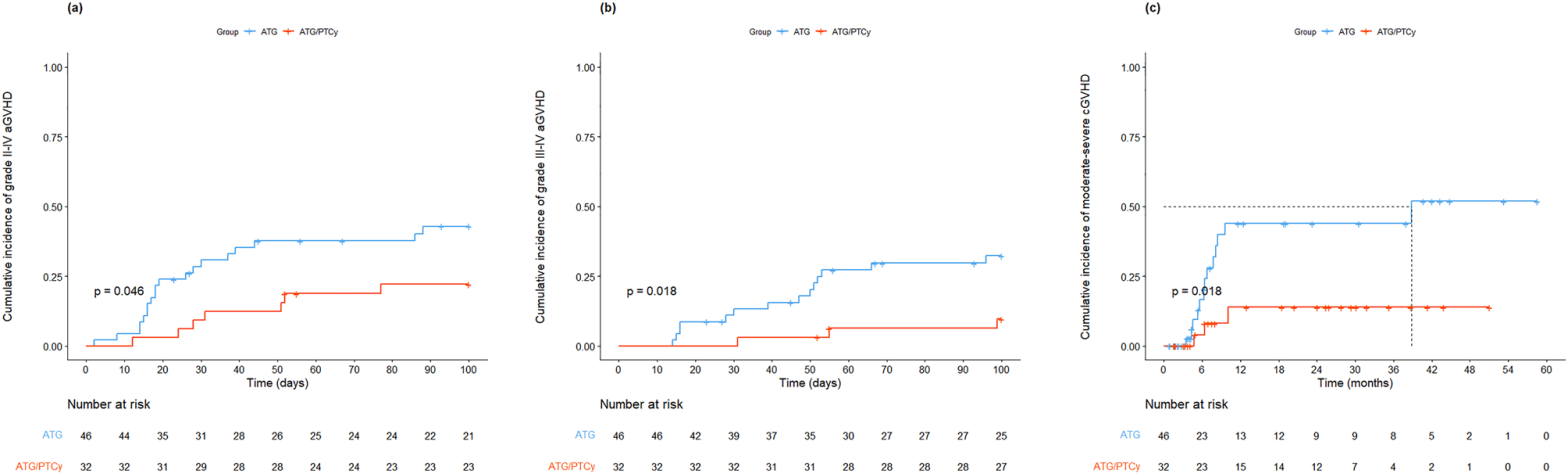
Graft-versus-host disease (GVHD) outcomes according to treatment groups. (**A**) Cumulative incidence of grade II-IV acute GVHD, (**B**) Cumulative incidence of grade III-IV acute GVHD, (**C**) Cumulative incidence of moderate-severe chronic GVHD.

The 100-day cumulative incidence of grade II-IV (22.1% vs. 42.9%, *P* = 0.046) and grade III-IV aGVHD (9.7% vs. 32.4%, *P* = 0.018), as well as 2-year moderate-severe cGVHD (13.9% vs. 43.9%, *P* = 0.018) in the ATG/PTCy group were significantly lower than those in the ATG group (Fig. 1, Table 2). The 2-year cumulative incidence of severe cGVHD in the ATG/PTCy group showed a trend toward decrease compared to that in the ATG group (6.3% vs. 28.4%, *P* = 0.072).

As presented in Table 3, the ATG/PTCy combination was a significantly protective factor for grade II-IV (HR 0.40; 95% CI, 0.17–0.96; *P* = 0.041) and grade III-IV (HR 0.21; 95% CI, 0.06–0.75; *P* = 0.016), as well as moderate-severe cGVHD (HR 0.25; 95% CI, 0.07–0.87; *P* = 0.029) in multivariate analysis. Patient age of ≤ 60 years was also associated with a lower grade III-IV aGVHD (HR 0.36; 95% CI, 0.14–0.95; *P* = 0.038).

**Table 3:** Multivariate analysis for transplantation outcomes.

	Adjusted HR	95% CI	*P*-value
Grade II-IV acute GVHD			
ATG/PTCy vs. ATG	0.40	0.17–0.96	0.041
CD34 cell count, ≤ 5.0 vs. > 5.0	0.56	0.25–1.24	0.152
Grade III-IV acute GVHD			
ATG/PTCy vs. ATG	0.21	0.06–0.75	0.016
Recipient Age, ≤ 60 vs. > 60	0.36	0.14–0.95	0.038
Moderate-severe chronic GVHD			
ATG/PTCy vs. ATG	0.25	0.07–0.87	0.029
GRFS			
ATG/PTCy vs. ATG	0.55	0.31–0.96	0.034
Diagnosis, MDS vs. Acute leukemia	0.62	0.33–1.16	0.136
Modified EBMT score, 1–3 vs. 4–6	0.61	0.34–1.08	0.089

*HR* hazard ratio, *CI* confidence interval, *GVHD* graft-versus-host disease, *ATG* anti-thymocyte globulin, *PTCy* post-transplant cyclophosphamide, *MDS* myelodysplastic syndrome, *EBMT* European Society for Blood and Marrow Transplantation, *GRF*S GVHD-free relapse-free survival.

### Survival outcomes

**Figure 2:**
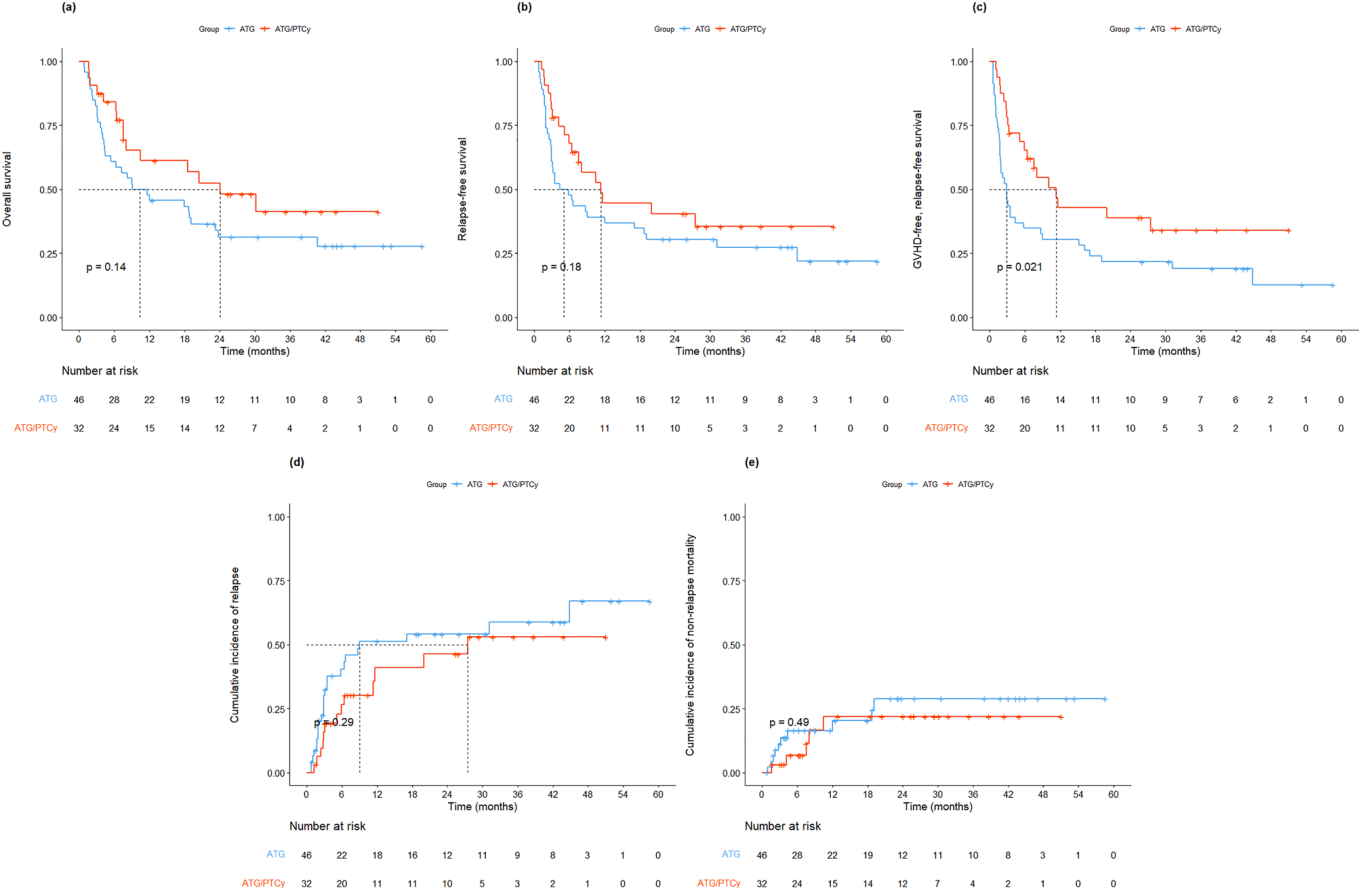
Survival outcomes according to treatment groups. (**A**) Kaplan–Meier curves of overall survival, (**B**) Kaplan–Meier curves of relapse-free survival, (**C**) Kaplan–Meier curves of GVHD-free, relapse-free survival, (**D**) Cumulative incidence of relapse, (E) Cumulative incidence of non-relapse mortality.

The median OS, RFS, and GRFS in the entire cohort were 18.5, 8.0, and 5.1 months, respectively. The 2-year probability of OS (52.5% vs. 31.3%, *P* = 0.14) and RFS (40.5% vs. 30.4%, *P* = 0.18) for ATG/PTCy and ATG groups were comparable (Fig. 2). The 2-year GRFS in the ATG/PTCy group was significantly higher compared to that in the ATG group (38.9% vs. 21.7%, *P* = 0.021). The univariate and multivariate analysis revealed that the GVHD prophylaxis protocol did not affect OS and RFS (Supplementary Table 1, Table 3). However, for GRFS, the ATG/PTCy protocol was an independent protective factor (HR 0.55; 95% CI, 0.31–0.96; *P* = 0.034). Additionally, a low mEBMT score (≤ 3) was marginally protective factor for GRFS compared to a high score (> 3) (HR 0.61; 95% CI, 0.34–1.08; *P* = 0.089).

The 2-year cumulative incidence of relapse and NRM in the entire cohort were 50.6% and 25.8%, respectively. The 2-year cumulative incidence of relapse (46.4% vs. 54.2%, *P* = 0.29) and NRM (21.9% vs. 29%, *P* = 0.49) were similar between the ATG/PTCy group and ATG group (Fig. 2).

### Infectious complications

Overall, 218 episodes of infections occurred in 66 of 78 patients (84.6%), with a median of 2 events/patient (mean, 2.8; range, 0–8). Detailed data of infectious events are summarized in Supplementary Table 2. 47.7% were of bacterial, 37.6% viral, and 14.7% fungal origin.

In pre-engraftment period (≤ day 30), there was no significant difference in the incidence of infection between the ATG/PTCy and ATG group (34.4% vs. 54.3%, *P* = 0.131) (Table 4). The 30-day cumulative incidences of severe bacterial, viral, and fungal infections were comparable between the groups (Supplementary Fig. 2). In the ATG/PTCy group, a lower proportion of patients experienced two or more infectious episodes compared to the ATG group (3.1% vs. 21.7%, *P* = 0.046).

**Table 4:** Infectious complications.

	Pre-engraftment (≤ 30 days)			Post-engraftment (> 30 days)		
	ATG/PTCy N = 32	ATG N = 46	*P*-value	ATG/PTCy N = 32	ATG N = 46	*P*-value
Infections, n (%)						
Severe	11 (34.4)	25 (54.3)	0.131	22 (68.8)	35 (76.1)	0.646
Life-threatening	1 (3.1)	5 (10.9)	0.406	7 (21.9)	24 (52.2)	0.014
Bacterial, n (%)						
Severe	4 (12.5)	12 (26.1)	0.239	15 (46.9)	26 (56.5)	0.543
Life-threatening	1 (3.1)	3 (6.5)	0.883	4 (12.5)	17 (37.0)	0.033
Viral, n (%)						
Severe	8 (25.0)	15 (32.6)	0.637	12 (37.5)	27 (58.7)	0.107
Life-threatening	0	1 (2.2)	1.000	0	8 (17.4)	0.035
Fungal, n (%)						
Severe	0	5 (10.9)	0.145	10 (31.2)	14 (30.4)	1.000
Life-threatening	0	1 (2.2)	1.000	3 (9.4)	6 (13.0)	0.890
Infections > 1, n (%)						
Severe	1 (3.1)	10 (21.7)	0.046	14 (43.8)	27 (58.7)	0.285
Life-threatening	0	1 (2.2)	1.000	1 (3.1)	10 (21.7)	0.046
Bacterial > 1, n (%)						
Severe	0	3 (6.5)	0.382	9 (28.1)	15 (32.6)	0.863
Life-threatening	0	1 (2.2)	1.000	1 (3.1)	5 (10.9)	0.406
Viral > 1, n (%)						
Severe	1 (3.1)	3 (6.5)	0.883	0	13 (28.3)	0.003
Life-threatening	0	0		0	1 (2.2)	1.000
Fungal > 1, n (%)						
Severe	0	0		0	3 (6.5)	0.382
Life-threatening	0	0		0	1 (2.2)	1.000

*ATG* anti-thymocyte globulin, *PTCy* post-transplant cyclophosphamide.

**Figure 3:**
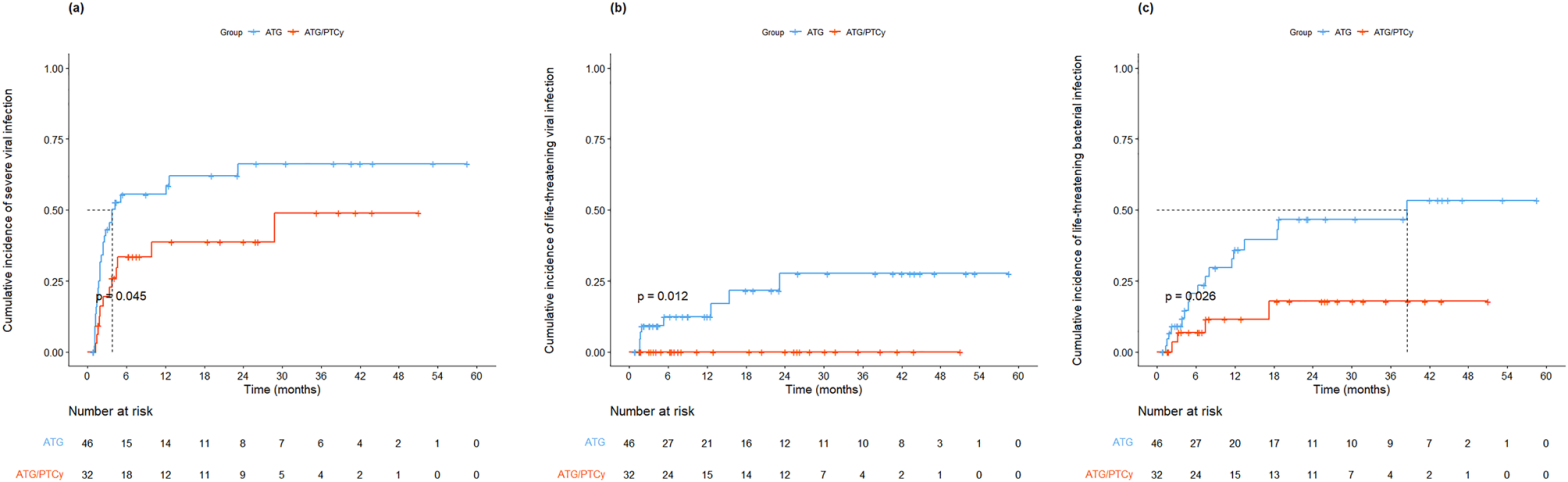
Cumulative incidence (CI) of post-engraftment infection according to treatment groups. (**A**) CI of severe viral infection, (**B**) CI of life-threatening viral infection, (**C**) CI of life-threatening bacterial infection.

In post-engraftment period (> day 30), the incidence of life-threatening bacterial (12.5% vs. 37%, *P* = 0.033) and viral infection (0% vs. 17.4%, *P* = 0.035) was lower in the ATG/PTCy group than the ATG group. The 1-year cumulative incidence of severe and life-threatening viral infection ([38.9% vs. 58.6%, *P* = 0.045], [0% vs. 12.4%, *P* = 0.012], respectively) and life-threatening bacterial infection (11.6% vs. 36.1%, *P* = 0.026) were significantly lower in the ATG/PTCy group compared to the ATG group (Fig. 3).

## Discussion

In patients with acute leukemia and MDS undergoing haplo-HSCT using PBSC and BuFlu conditioning, this study demonstrated the efficacy of our novel GVHD prophylaxis protocol (combining low-dose PTCy and ATG adjusted for days -3 ALC levels) in reducing severe GVHD compared to the standard ATG-based regimen. Dual T-cell depletion with individually tailored ATG and attenuated dose of PTCy did not elevate relapse risk and improved GRFS. Additionally, ATG/PTCy resulted in fewer severe infections than ATG.

Due to PTCy’s potential to enhance the effect of ATG in mitigating GVHD^17^, recent studies have explored various combinations of ATG and PTCy to improve GVHD prevention in haplo-HSCT (Supplementary Table 3). The cumulative incidence of aGVHD with ATG/PTCy in our study was comparable with other reports, indicating 11.5–34.6% for grade II-IV and 5–9% for grade III-IV aGVHD^25–28^. These studies consistently suggest that, despite variations in dosing regimens, dual T-cell depletion with ATG/PTCy can be more effective in reducing severe aGVHD than ATG alone. Additionally, we found a significantly lower cumulative incidence of moderate-severe cGVHD for ATG/PTCy. As the mechanisms of GVHD prevention differ between ATG and PTCy^29,30^, the synergistic effect of the ATG/PTCy combination can be influenced by the dosage and timing of each. Studies with PTCy at a total dose of only 29 mg/kg^26^ and ATG used post-transplant at a dose of 2.5 mg/kg^25^ showed no difference in the incidence of moderate-severe cGVHD between the two groups. In contrast, a study using a total PTCy dose of 80 mg/kg (with a total of 7.5 mg/kg of pre-transplant ATG), similar to ours, demonstrated a fewer extensive cGVHD in the ATG/PTCy group^27^. Furthermore, in a study using ATG 5 mg/kg and PTCy 50 mg/kg, the ATG/PTCy group had less moderate-severe cGVHD^31^, consistent with our findings. In our study, ATG/PTCy significantly reduced GVHD but did not impact the relapse risk. Instead, we observed a significantly higher GRFS in the ATG/PTCy group, consistent with previous studies^25,26^. Our result demonstrated that dual T-cell depletion with ATG/PTCy effectively reduces the risk of severe GVHD without increasing the relapse risk, resulting in a favorable GRFS outcome. Further mechanism studies on the dosing of ATG and PTCy are warranted to achieve the optimal synergistic effect of ATG/PTCy protocol.

Excessive exposure to ATG may increase immunosuppressive toxicity, elevating risks of infections and relapse^32^. This risk could be heightened when used with PTCy, emphasizing the need for a strategy to judiciously reduce the dose of ATG^26^. Previous studies reported that weight-based dosing of ATG carries the risk of overdose, and the ALC level on the day of ATG administration could optimize ATG dosing^18,33^. Our ATG/PTCy protocol tailored the dose of ATG individually for each recipient based on the ALC level at day -3; i.e., if the ALC is low, a small amount of ATG is administered. The combination of low-dose ATG and PTCy did not increase CMV infection compared to the standard dose of ATG, consistent with previous studies^25,31^. Additionally, we found a significant decrease in life-threatening bacterial and viral infection in the ATG/PTCy group, particularly in the post-engraftment period. As ATG is associated with a delay of the immune reconstitution, an attenuated dose of ATG might ameliorate post-engraftment viral infections^34^. Moreover, the ATG group exhibited more moderate-severe cGHVD, suggesting the potential for prolonged immunosuppressive therapy. These factors could have contributed to a higher incidence of bacterial infections in the ATG group. The reduced risk of life-threatening infections might have been more attributed to individually tailored ATG dosing than to the synergistic effect of ATG and PTCy. Further research is needed on personalized dosing in GVHD prophylaxis with ATG/PTCy for haplo-HSCT.

This study had several limitations. First, this is a retrospective study. Thus, the two groups were not randomized and characteristics between the two groups were not fully comparable, although generally well-balanced. Although ATG/PTCy was identified as an independently favorable factor in the multivariate analyses, there is a possibility of confounding effects due to patient selection and differences between the groups, such as the use of additional immunosuppressants and timing of HSCT. Second, a relatively small sample size may limit the statistical power. Instead, we minimized the confounding factors; diseases included only MDS and acute leukemia, and all patients received an identical conditioning regimen and graft source, and infection prophylaxis. Third, we did not investigate data on immune reconstitution and ATG concentration. For the optimal dose and schedule of ATG/PTCy, further mechanism studies are warranted.

Despite these limitations, our study implemented a novel personalized dosing strategy by combining low-dose PTCy with a reduced dose of ATG tailored to the ALC level on the day of ATG infusion. We compared its outcomes with those of an ATG-based regimen in haplo-HSCT with the same graft source and conditioning regimen. Additionally, we conducted a comparative analysis of infection episodes, identifying differences between the two groups in terms of timing and etiology of infections.

In conclusion, our results demonstrated that dual T-cell depletion with an individually tailored ATG and attenuated dose of PTCy led to a low incidence of both severe aGVHD and cGVHD, improving GRFS compared to the standard-dose ATG regimen. In addition, ATG/PTCy showed a low incidence of severe infections, making it a feasible protocol. Reducing the PTCy dosage slightly and incorporating an individually tailored small amount of ATG appears to be a promising strategy in haplo-HSCT, emphasizing the need for further research to determine the optimal regimen.

### Supplementary Information

Supplementary Information.

## Data Availability

The datasets generated during the current study are available from the corresponding authors on reasonable request.
