# Supplementary material for: Dual T-cell depletion with individually tailored anti-thymocyte globulin and attenuated dose of post-transplant cyclophosphamide in haploidentical peripheral stem cell transplantation
Source: Sci Rep. 2024 Jun 16;14:13885. doi: 10.1038/s41598-024-64361-5 (PMC11180652; doi:10.1038/s41598-024-64361-5)
Supplement: Supplementary file 1 — Supplementary Information. [file 41598_2024_64361_MOESM1_ESM.docx]

**Supplementary Figure 1:** Scheme of GVHD prophylaxis regimen**.**

**
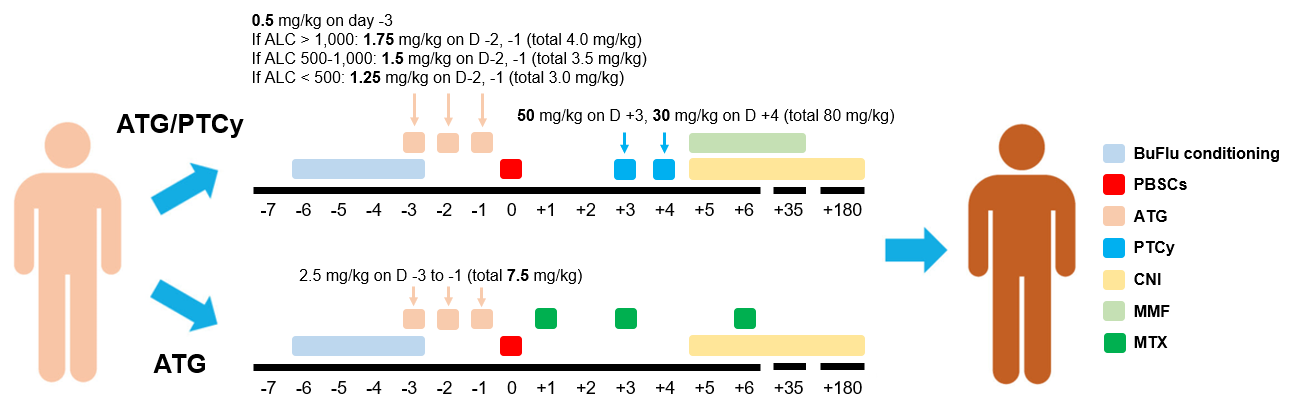
**

Cyclosporine was initiated at 3mg/kg 48 hours before stem cell infusion, with dose adjustments made to attain the target serum trough level of 250-400 ng/ml. Tacrolimus was started at 0.04 mg/kg on day 5, with adjustments to achieve the target level of 5-15 ng/ml.

**Supplementary Figure 2:** Cumulative incidence of pre-engraftment infection according to treatment groups; (A) bacterial infection; (B) viral infection; (C) fungal infection.


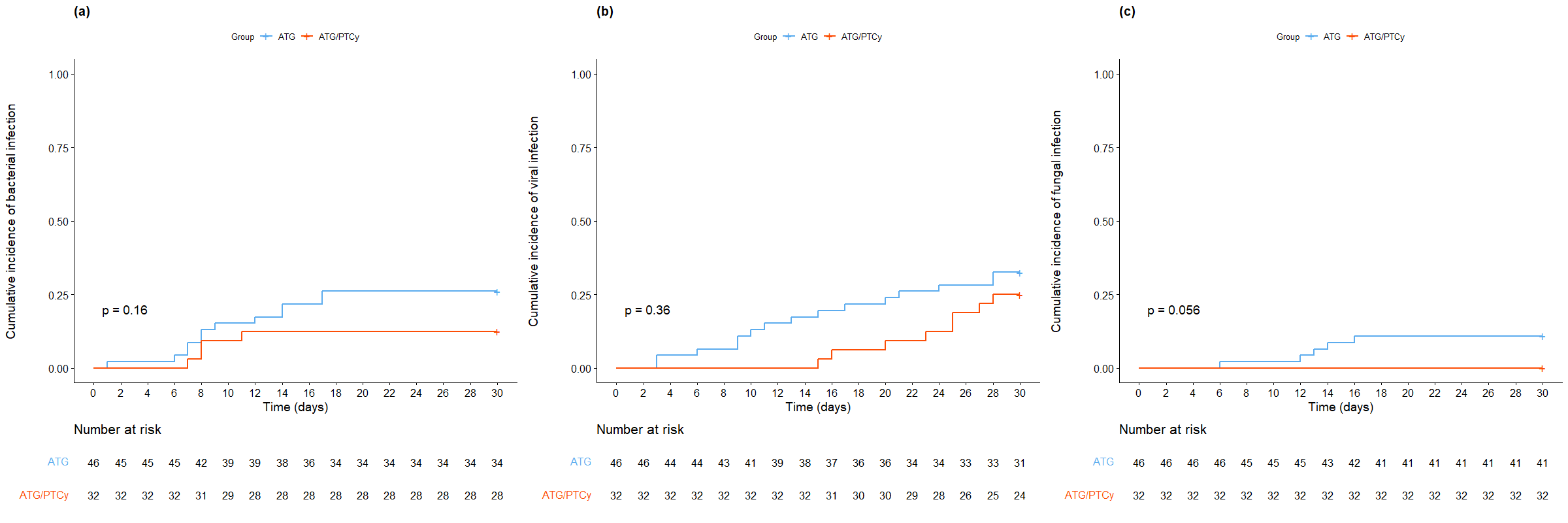


**Supplementary Table 1:** Univariate analysis for transplantation outcomes.

|  | **HR** | **95% CI** | ***P*-value** |
| --- | --- | --- | --- |
| **GVHD-related** |  |  |  |
| **Grade Ⅱ-Ⅳ acute GVHD**  ATG/PTCy vs. ATG  Recipient Age, ≤ 60 vs. > 60  Diagnosis, MDS vs. Acute leukemia  Modified EBMT score, 1-3 vs. 4-6  Conditioning intensity, RIC vs. MAC  CD34 cell count, ≤ 5.0 vs. > 5.0  HSCT timing, until June 2020 vs. after June 2020 | 0.42  0.80  1.31  0.63  1.01  0.60  1.32 | 0.18-1.01  0.36-1.81  0.58-2.93  0.27-1.44  0.38-2.67  0.27-1.33  0.60-2.92 | 0.053  0.597  0.517  0.273  0.990  0.209  0.485 |
| **Grade IⅡ-Ⅳ acute GVHD**  ATG/PTCy vs. ATG  Recipient Age, ≤ 60 vs. > 60  Diagnosis, MDS vs. Acute leukemia  Modified EBMT score, 1-3 vs. 4-6  Conditioning intensity, RIC vs. MAC  CD34 cell count, ≤ 5.0 vs. > 5.0  HSCT timing, until June 2020 vs. after June 2020 | 0.25  0.44  0.97  0.44  1.15  0.55  2.08 | 0.07-0.86  0.17-1.14  0.34-2.74  0.14-1.35  0.33-4.00  0.20-1.48  0.73-5.90 | 0.028  0.090  0.949  0.153  0.829  0.233  0.169 |
| **Moderate-severe chronic GVHD**  ATG/PTCy vs. ATG  Recipient Age, ≤ 60 vs. > 60  Diagnosis, MDS vs. Acute leukemia  Modified EBMT score, 1-3 vs. 4-6  Conditioning intensity, RIC vs. MAC  CD34 cell count, ≤ 5.0 vs. > 5.0  HSCT timing, until June 2020 vs. after June 2020 | 0.25  1.35  1.48  0.80  1.31  1.50  0.83 | 0.07-0.87  0.43-4.20  0.54-4.10  0.29-2.20  0.30-5.79  0.56-4.04  0.30-2.26 | 0.029  0.603  0.445  0.662  0.720  0.424  0.716 |
| **Survival-related** |  |  |  |
| **OS**  ATG/PTCy vs. ATG  Recipient Age, ≤ 60 vs. > 60  Diagnosis, MDS vs. Acute leukemia  Modified EBMT score, 1-3 vs. 4-6  Conditioning intensity, RIC vs. MAC  CD34 cell count, ≤ 5.0 vs. > 5.0  HSCT timing, until June 2020 vs. after June 2020 | 0.64  1.03  0.98  0.83  1.00  1.08  1.23 | 0.34-1.17  0.55-1.93  0.52-1.83  0.46-1.51  0.44-2.24  0.61-1.91  0.68-2.24 | 0.148  0.919  0.938  0.540  0.994  0.793  0.495 |
| **RFS**  ATG/PTCy vs. ATG  Recipient Age, ≤ 60 vs. > 60  Diagnosis, MDS vs. Acute leukemia  Modified EBMT score, 1-3 vs. 4-6  Conditioning intensity, RIC vs. MAC  CD34 cell count, ≤ 5.0 vs. > 5.0  HSCT timing, until June 2020 vs. after June 2020 | 0.67  1.21  0.75  0.75  0.89  1.29  1.33 | 0.38-1.19  0.66-2.21  0.40-1.40  0.42-1.33  0.43-1.83  0.75-2.22  0.75-2.35 | 0.176  0.537  0.361  0.329  0.743  0.365  0.328 |
| **GRFS**  ATG/PTCy vs. ATG  Recipient Age, ≤ 60 vs. > 60  Diagnosis, MDS vs. Acute leukemia  Modified EBMT score, 1-3 vs. 4-6  Conditioning intensity, RIC vs. MAC  CD34 cell count, ≤ 5.0 vs. > 5.0  HSCT timing, until June 2020 vs. after June 2020 | 0.53  0.93  0.72  0.63  1.15  1.11  1.31 | 0.30-0.91  0.53-1.63  0.39-1.31  0.36-1.10  0.56-2.35  0.66-1.87  0.76-2.24 | 0.023  0.797  0.281  0.103  0.707  0.703  0.330 |

HR, hazard ratio; CI, confidence interval; GVHD, graft-versus-host disease; ATG, anti-thymocyte globulin; PTCy, post-transplant cyclophosphamide; MDS, myelodysplastic syndrome; EBMT, European Society for Blood and Marrow Transplantation; RIC, reduced-intensity conditioning; MAC, myeloablative conditioning; GRFS, GVHD-free, relapse-free survival; OS, overall survival; RFS, relapse-free survival.

**Supplementary Table 2:** Details of infectious episodes.

|  | **Pre-engraftment (≤ 30 days)** | | **Post-engraftment (> 30 days)** | |
| --- | --- | --- | --- | --- |
|  | **ATG/PTCy** | **ATG** | **ATG/PTCy** | **ATG** |
| **Total severe infections** | **N = 13** | **N = 39** | **N = 51** | **N = 115** |
| Life-threatening | 1 | 6 | 8 | 39 |
| **Bacterial infection**  Life-threatening | N = 4  1 | N = 16  4 | N = 29  5 | N = 55  23 |
| **viral infection**  Life-threatening | N = 9  0 | N = 18  1 | N = 12  0 | N = 43  9 |
| **Fungal infection**  Life-threatening | N = 0  0 | N = 5  1 | N = 10  3 | N = 17  7 |
| **Infection etiology** |  |  |  |  |
| **Bacterial**  **Gram-positive**  Staphylococcus spp  Streptococcus spp  Enterococcus spp  Corynebacterium spp  Others  **Gram-negative**  Escherichia coli  Klebsiella spp  Pseudomonas aeruginosa  Stenotrophomonas maltophilia  Acinetobacter baumannii  Enterobacter cloacae  Others | N = 4  0  0  0  2  0  1  1  0  0  0  0  0 | N = 16  2  0  3  3  1  1  2  2  1  1  0  0 | N = 29  2  0  8  4  1  2  4  3  0  0  1  4 | N = 55  12  1  17  6  3  7  3  1  0  1  3  1 |
|  |  |  |  |  |
| **Viral**  Cytomegalovirus  Epstein-Barr virus  Herpes simplex virus  Varicella-zoster virus  BK Poliomavirus  Parainfluenza virus  Respiratory syncytial virus  COVID-19 | N = 9  7  0  0  0  1  1  0  0 | N = 18  9  0  8  0  0  0  1  0 | N = 12  10  0  0  1  0  0  0  1 | N = 43  24  2  9  4  2  0  1  1 |
|  |  |  |  |  |
| **Fungal**  Pneumocystis jirovecii  Candida spp  Aspergillosis spp  Mucor spp | N = 0  0  0  0  0 | N = 5  0  2  2  1 | N = 10  2  2  6  0 | N = 17  0  5  12  0 |
| **Infection site, N**  Bloodstream infection  Catheter-related infection  Pneumonia  CMV reactivation  Urinary tract infection  Others | 4  0  0  7  1  1 | 10  3  6  8  0  12 | 20  7  13  9  0  2 | 31  18  17  17  6  26 |

ATG, anti-thymocyte globulin; PTCy, post-transplant cyclophosphamide; CMV, cytomegalovirus.

**Supplementary Table 3:** Summary of outcome from the literature regarding the ATG/PTCy combination for haplo-PBSCT.

|  | **Patient** | **intensity** | **ATG/PTCy regimen** | **Acute GVHD** | **Chronic GVHD** | **Survival** |
| --- | --- | --- | --- | --- | --- | --- |
| **Our study** | N = 32  Acute leukemia, MDS  Median age 56 | MAC/RIC  BuFlu | ATG total 3-4 mg/kg (adjusted for D-3 ALC)  PTCy total 80 mg/kg | **CI at day 100**  Any 38.6%  Gr 2-4 22.1%  Gr 3-4 9.7% | **CI at 2 year**  Any 46.3%  Mod-sev 13.9%  Sev 6.3% | **At 2 year**  OS 52.5%  RFS 40.5%  GRFS 38.9% |
|  |  |  |  |  |  |  |
| **2018 Law et al.** | N = 50  All disease  Median age 56 | RIC  BuFlu+TBI | ATG total 4.5 mg/kg  PTCy total 100 mg/kg | **CI at day 100**  Any 38.3%  Gr 2-4 20.3%  Gr 3-4 5.2% | **CI at 6 month**  Any 15.5%  Sev 0% | **At 1 year**  OS 48.1%  RFS 35.7% |
|  |  |  |  |  |  |  |
| **2019 Yang et al.** | N = 32  All disease  Median age 37 | MAC/RIC  BuFlu+Ara-C,  TBI+Cy+etoposide | ATG total 5.0 mg/kg  PTCy total 50 mg/kg | **CI at day 100**  Gr 2-4 19.4%  Gr 3-4 6.9% | **CI at 6 month**  Mod-sev 18.8% | **At 1 year**  OS 78.4%  DFS 59% |
|  |  |  |  |  |  |  |
| **2019 Wang et al.** | N = 114  All disease  Median age 27 | MAC  “Beijing protocol” | ATG total 10 mg/kg  PTCy total 29 mg/kg | **CI at day 100**  Gr 2-4 26%  Gr 3-4 5% | **CI at 2 year**  Any 30%  Mod-sev 17% | **At 2 year**  OS 83%  DFS 81%  GRFS 63% |
|  |  |  |  |  |  |  |
| **2022 Barkhordar et al.** | N = 78  AML or ALL  Median age 27.5 | MAC  BuCy | ATG total 7.5 mg/kg  PTCy 80 mg/kg | **CI at day 100**  Gr 2-4 34.6%  Gr 3-4 8.97% | **CI at 1 year**  Extensive 13.6% | **At 1 year**  OS 66.7%  DFS 60.3%  GRFS 48.7% |
|  |  |  |  |  |  |  |
| **2022 Zhang et al.** | N = 61  All disease  Median age 24 | MAC  BuFlu+Ara-C | ATG total 2.5 mg/kg  PTCy 80 mg/kg | **CI at day 100**  Gr 2-4 11.5%  Gr 3-4 6.6% | **CI at 2 year**  Any 24.2%  Mod-sev 14.5% | **At 2 year**  OS 75.4%  DFS 72.7%  GRFS 61.3% |

ATG, antithymocyte globulin; PTCy, post-transplant cyclophosphamide; GVHD, graft-versus-host disease; MDS, myelodysplastic syndrome; MAC, myeloablative conditioning; RIC, reduced-intensity conditioning; Bu, busulfan; Flu, fludarabine; ALC, absolute lymphocyte count; CI, cumulative incidence; OS, overall survival; RFS, relapse-free survival; GRFS, GVHD-free, relapse-free survival; TBI, total body irradiation; Cy, cyclophosphamide; DFS, disease-free survival.
